# Structure of *Pseudomonas aeruginosa* inosine 5′-monophosphate dehydrogenase

**DOI:** 10.1107/S1744309113002352

**Published:** 2013-02-22

**Authors:** Vincenzo A. Rao, Sharon M. Shepherd, Richard Owen, William N. Hunter

**Affiliations:** a Division of Biological Chemistry and Drug Discovery, College of Life Sciences, University of Dundee, Dow Street, Dundee DD1 5EH, Scotland

**Keywords:** inosine 5′-monophosphate dehydrogenase, *Pseudomonas aeruginosa*, antimicrobial drug targets

## Abstract

Inosine 5′-monophosphate dehydrogenase (IMPDH) represents a potential antimicrobial drug target. The crystal structure of recombinant *Pseudomonas aeruginosa* IMPDH has been determined to a resolution of 2.25 Å. The structure is a homotetramer of subunits dominated by a (β/α)_8_-barrel fold, consistent with other known structures of IMPDH. Also in common with previous work, the cystathionine β-synthase domains, residues 92–204, are not present in the model owing to disorder. However, unlike the majority of available structures, clearly defined electron density exists for a loop that creates part of the active site. This loop, composed of residues 297–315, links α8 and β9 and carries the catalytic Cys304. *P. aeruginosa* IMPDH shares a high level of sequence identity with bacterial and protozoan homologues, with residues involved in binding substrate and the NAD^+^ cofactor being conserved. Specific differences that have been proven to contribute to selectivity against the human enzyme in a study of *Cryptosporidium parvum* IMPDH are also conserved, highlighting the potential value of IMPDH as a drug target.

## Abstract 2

The crystal structure of inosine 5′-monophosphate dehydrogenase from *P. aeruginosa* has been determined to 2.25 Å resolution.

## 1. Introduction

Inosine 5′-monophosphate dehydrogenase (IMPDH; EC 1.1.1.205) catalyzes the first committed reaction in *de novo* guanine-nucleotide biosynthesis. This enzyme converts inosine 5′-monophosphate (IMP) to xanthosine 5′-monophosphate (XMP), which can then be converted to guanosine 5′-monophosphate (GMP) by GMP synthetase. Guanine nucleotides are essential for signal transduction and DNA/RNA synthesis and IMPDH therefore plays a pivotal role in the growth and proliferation of both prokaryotic and eukaryotic cells (Hedstrom, 2009 ▶). The enzyme has been the subject of thorough kinetic and crystallographic studies. Structures of IMPDH from a variety of organisms are known, including pathogenic bacteria such as *Borrelia burgdorferi* (McMillan *et al.*, 2000 ▶; PDB entry 1eep), *Bacillus anthracis* (Center for Structural Genomics of Infectious Diseases, unpublished work; PDB entry 3tsb) and *Streptococcus pyogenes* (Zhang *et al.*, 1999 ▶), the protozoan parasites *Cryptosporidium parvum* (MacPherson *et al.*, 2010 ▶; PDB entry 3ffs) and *Tritrichomonas foetus* (Whitby *et al.*, 1997 ▶; PDB entry 1ak5), and human type II (Colby *et al.*, 1999 ▶; PDB entry 1b30).

The enzyme mechanism is thought to start with a catalytic cysteine attacking the C2 position of IMP; an NAD^+^-dependent dehydro­genation then generates NADH and the covalent product intermediate E-XMP*. The expulsion of NADH promotes the insertion of a mobile flap to occupy the vacated cofactor-binding site. An arginine then acts as a general base catalyst and supports hydrolysis to convert E-XMP* into XMP and to recharge the enzyme (Hedstrom, 2009 ▶). IMPDH has been identified as a potential drug target for antimicrobial infections (Hedstrom *et al.*, 2011 ▶). Progress has been made in developing highly potent inhibitors of the enzyme from *C. parvum*, which also display antiparasitic efficacy against *Toxoplasma gondii* (Gorla *et al.*, 2012 ▶). Critically, these researchers have also addressed the issue of selectivity over the human homologue and a selectivity of greater than 1000-fold has been attained for the microbial enzymes.

Our interest is in the opportunistic Gram-negative pathogen *Pseudomonas aeruginosa*, which is widely recognized as a significant cause of hospital-acquired infections that often affect patients with compromised immune systems (Gaynes & Edwards, 2005 ▶). As part of a wide-ranging project to advance early-stage drug discovery against *P. aeruginosa* (Eadsforth *et al.*, 2012 ▶; Moynie *et al.*, 2013 ▶), we have adopted a structure-based approach (Hunter, 2009 ▶) to drive target assessment. Genetic evidence exists suggesting that the gene encoding IMPDH is essential in several bacteria, including *P. aeruginosa* (Liberati *et al.*, 2006 ▶). We therefore sought to generate a recombinant source of the enzyme and to investigate its structure. Here, we report success in this endeavour and present the crystal structure of apo IMPDH from *P. aeruginosa* at 2.25 Å resolution. Comparisons with the structures of the enzyme from *C. parvum* (*Cp*IMPDH) and *Homo sapiens* suggests that it may be possible to selectively inhibit the bacterial IMPDH over that of the host.

## 2. Methods

### 2.1. Protein expression and purification

The full-length gene (PA3770) for *P. aeruginosa* IMPDH (*Pa*IMPDH; amino acids 1–489; UniProt ID Q9HXM5) was synthesized and codon-optimized for expression in *Escherichia coli* (GenScript). It was cloned into a modified pET15b (Novagen) cloning vector which encodes an N-terminal His tag followed by a tobacco etch virus (TEV) protease cleavage site and the protein of interest (Rao *et al.*, 2011 ▶). The integrity of the construct was verified by DNA sequencing (DNA Sequencing Unit, University of Dundee). Recombinant *Pa*IMPDH was produced in *E. coli* BL21 (DE3) pLysS cells (Stratagene) at 310 K in LB medium supplemented with 100 µg ml^−1^ ampicillin. The cells were grown to an OD_600_ of ∼0.7 before induction with isopropyl β-d-1-thiogalactopyranoside at a final concentration of 1 m*M* and growth at 293 K for a further 24 h. The cells were harvested by centrifugation (3500*g* at 277 K for 40 min). The cell pellet was resuspended in buffer (25 m*M* Tris–HCl pH 8.5, 500 m*M* NaCl, 20 m*M* imidazole) supplemented with an EDTA-free protease-inhibitor cocktail tablet (Calbiochem. The cells were lysed by passage through a continuous-flow cell disruptor (Constant Systems) at 207 MPa and the cell debris was removed following centrifugation (40 000*g* at 277 K for 20 min). *Pa*IMPDH was purified using affinity chromatography on a 5 ml HisTrap HP column (GE Healthcare) pre-charged with Ni^2+^ and equilibrated in buffer *A* (25 m*M* Tris–HCl pH 8.5, 500 m*M* NaCl, 20 m*M* imidazole). A linear concentration gradient of imidazole was applied using buffer *B* (25 m*M* Tris–HCl pH 8.5, 500 m*M* NaCl, 500 m*M* imidazole) to elute the protein, which was then dialyzed against buffer *C* (25 m*M* Tris–HCl pH 7.5, 250 m*M* NaCl) at 277 K. Fractions were analyzed using SDS–PAGE and those containing *Pa*IMPDH were pooled. The protein was further purified, with the His tag still present, by size-exclusion chromatography using a Superdex 75 26/60 column (GE Healthcare) equilibrated with buffer *C* on an ÄKTApurifier (GE Healthcare). This column had previously been calibrated with molecular-weight standards: blue dextran (>2000 kDa), thyroglobulin (669 kDa), ferritin (440 kDa), aldolase (158 kDa), conalbumin (75 kDa), ovalbumin (43 kDa), carbonic anhydrase (29.5 kDa), ribonuclease A (13.7 kDa) and aprotinin (6.5 kDa) (GE Healthcare; data not shown). The protein eluted as a single species of approximate mass 200 kDa. Fractions containing the protein were pooled and concentrated to 22 mg ml^−1^ using Amicon Ultra devices (Millipore) for subsequent use. A final yield of 25 mg per litre of bacterial culture was obtained. The purity of the protein was confirmed by SDS–PAGE and mass spectrometry (Fingerprint Proteomics Facility, University of Dundee). A theoretical extinction coefficient of 22 450 *M* ^−1^ cm^−1^ at 280 nm was used to estimate the protein concentration (*ProtParam*; Gasteiger *et al.*, 2005 ▶); the theoretical mass of one subunit was estimated as 54 kDa, with a calculated isoelectric point of 6.8. The purified protein was stored at 277 K in buffer *C* for subsequent use.

### 2.2. Crystallization, data collection and structure determination

A variety of commercial screens were employed to identify appropriate starting conditions for crystal growth. Diffraction-quality crystals of *Pa*IMPDH were obtained at room temperature by sitting-drop vapour diffusion with a reservoir solution consisting of 0.2 *M* sodium acetate pH 4.5, 40% 2-methyl-2,4-pentanediol (MPD). Prismatic crystals appeared after a month from a mixture of equal volumes of protein solution (9 mg ml^−1^ in 25 m*M* Tris–HCl pH 7.5, 250 m*M* NaCl from the stock protein solution) and reservoir solution. The concentration of protein in the drop is therefore about 4.5 mg ml^−1^. The crystals grew to a maximum dimension of approximately 0.15 mm. The high concentration of MPD in the reservoir solution proved to be a suitable cryoprotectant and data were collected at 100 K using a Rigaku MicroMax-007 rotating-anode X-­ray generator (Cu *K*α, λ = 1.5418 Å) coupled to an R-AXIS IV^++^ image-plate detector. The data were indexed with *XDS* (Kabsch, 2010 ▶) and scaled using *SCALA* (Evans, 2006 ▶) from the *CCP*4 program suite (Winn *et al.*, 2011 ▶). The structure was solved by molecular replacement with *Phaser* (McCoy *et al.*, 2007 ▶) using the coordinates of a monomer (chain *A*) of IMPDH from *B. anthracis* (PDB entry 3tsb; Center for Structural Genomics of Infectious Diseases, unpublished work) as a search model. The search model shares 54% sequence identity with *Pa*IMPDH. Model building and manipulation of the model were carried out using *Coot* (Emsley *et al.*, 2010 ▶). Refinement calculations were performed using *REFMAC*5 (Murshudov *et al.*, 2011 ▶) and translation/libration/screw analysis (TLS) was applied (Painter & Merritt, 2006 ▶). The refinement proceeded with the incorporation of water molecules following conservative criteria (Leonard & Hunter, 1993 ▶), a chloride and a number of side chains with dual conformers. The refinement was terminated when there were no significant changes in the *R*_work_ and *R*_free_ values and when inspection of the difference map suggested that no further corrections or additions were required. The stereochemistry and quality of the model were validated using *MolProbity* (Chen *et al.*, 2010 ▶). Data-collection and structure-refinement statistics are shown in Table 1 ▶.

## 3. Results and discussion

### 3.1. Structure determination

A highly efficient recombinant protein-expression system has been prepared and protocols for purification and crystallization have been established. The crystal structure of *Pa*IMPDH was determined at 2.25 Å resolution. The asymmetric unit consists of a single polypeptide chain comprising residues 1–91, 205–370 and 426–467, with an estimated solvent content of 60% and a *V*_M_ (Matthews, 1968 ▶) of 2.94 Å^3^ Da^−1^. The initial model consisted of 261 residues, with a correlation coefficient of 0.75 and *R*_work_ and *R*_free_ values of 37.2% and 41.8%, respectively. Subsequent model building and refinement extended this to 293 residues, with a correlation coefficient of 0.96 and improved *R*_work_ and *R*_free_ values of 14.8% and 19.0%, respectively. Analysis of the Ramachandran plot revealed that 99.3% of the residues were in the allowed region, with only two outliers: Gly226 and Gln458. These two residues are located on flexible loops linking α8 to β9 and α13 to β12, respectively, and the associated electron density is relatively poor. A greater degree of disorder was evident at several positions, where it was not even possible to interpret the electron density. Consequently, 196 residues are absent from the final model. A further comment on this will be made below.

### 3.2. Overall structure

The crystal structures of IMPDHs from several species have previously been determined and an excellent review has been published (Hedstrom, 2009 ▶). In brief, IMPDH forms homotetramers. The subunit is constructed from two segments: a catalytic domain which displays the (β/α)_8_-barrel fold and a subdomain containing two cystathionine β-­synthase (CBS)/Bateman domains (Bateman, 1997 ▶; Hedstrom, 2009 ▶). The function of the CBS domains in this enzyme system is a source of debate (Hedstrom *et al.*, 2011 ▶). It has been determined that the deletion of these subdomains has no effect on the enzymatic activity (Nimmesgern *et al.*, 1999 ▶). This subdomain protrudes out from the catalytic domain and is usually disordered in IMPDH structures (Hedstrom, 2009 ▶).

**Figure 1:**
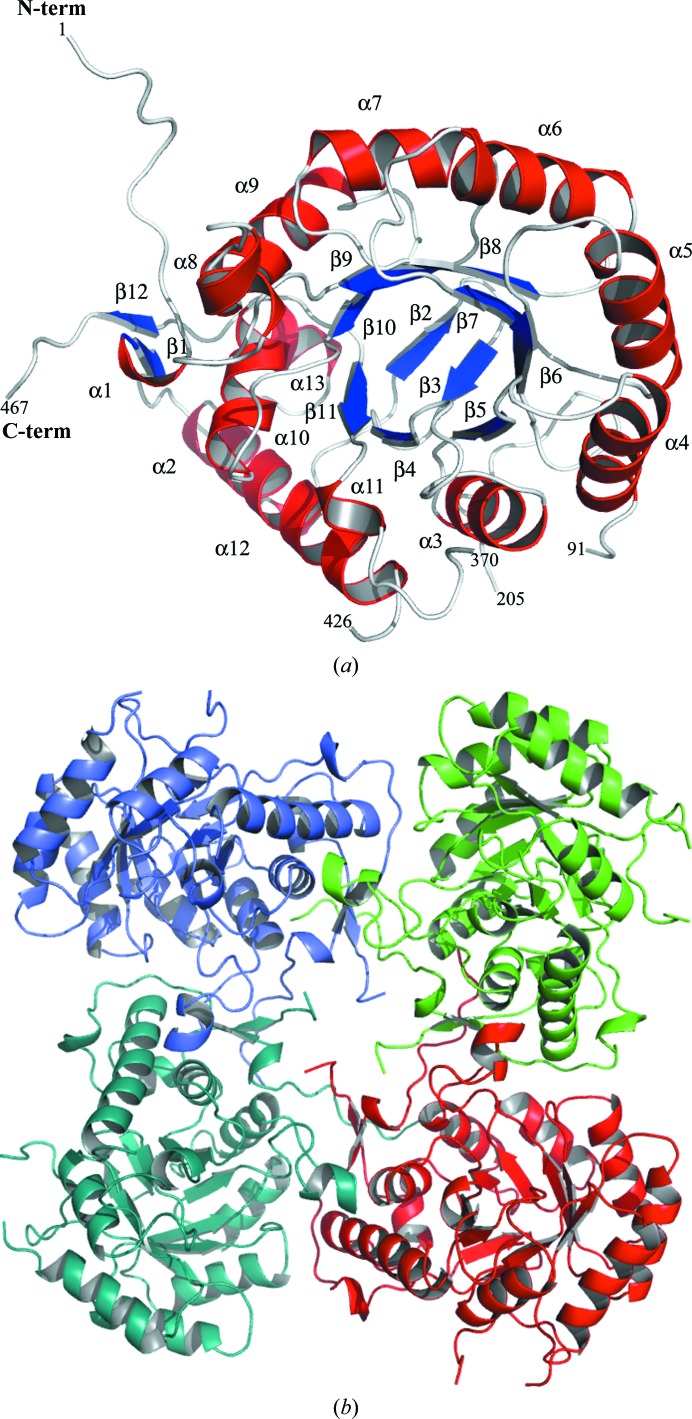
(*a*) The crystal structure of *Pa*IMPDH displays a (β/α)_8_-barrel fold. β-Strands and α-helices are coloured blue and red, respectively. (*b*) View parallel to the crystallographic fourfold axis showing the *Pa*IMPDH tetramer.

**Figure 2:**
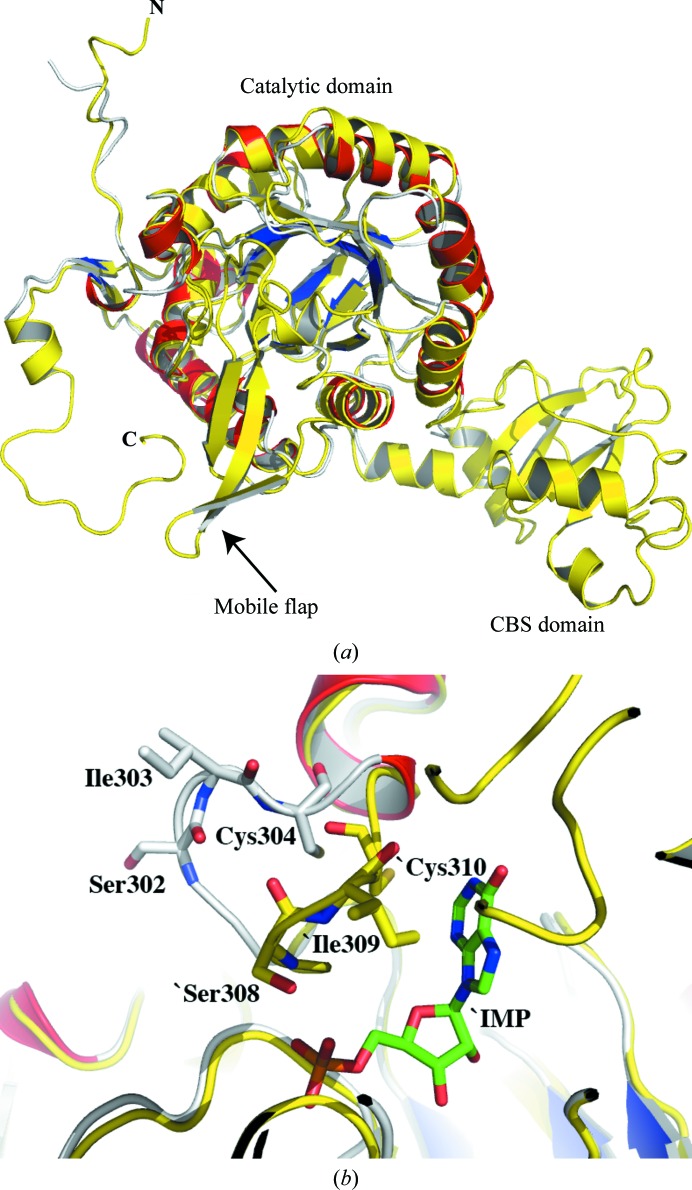
(*a*) Superposition of *Pa*IMPDH (grey main-chain trace with red helices and blue strands) and *S. pyogenes* IMPDH (all yellow; PDB entry 1zfj) matches 293 residues with an r.m.s.d. of 1.1 Å based on a least-squares fit of C^α^ positions and highlights the missing residues in *Pa*IMPDH as corresponding to the CBS subdomain, a mobile flap and C-terminal regions. (*b*) Close-up of the active site. The active-site loop of *Pa*IMPDH (grey) is fully ordered and adopts an ‘open’ conformation compared with the ‘closed’ conformation observed when a ligand is bound to the *S. pyogenes* enzyme (yellow). The distances from the catalytic cysteine S^γ^ atoms to IMP C2 are approximately 6 and 3 Å in *Pa*IMPDH and *Sp*IMPDH, respectively.

*Pa*IMPDH displays the canonical (β/α)_8_-barrel fold of the catalytic domain, with approximate dimensions of 40 × 40 × 50 Å (Fig. 1 ▶ *a*). One subunit comprises the asymmetric unit and the tetramer is formed by the crystallographic fourfold axis with symmetry operators −*y*, *x*, *z*; −*x* + 1/2, −*y* + 1/2, *z* − 1/2 and −*y* + 1/2, *x* − 1/2, *z* − 1/2 (Fig. 1 ▶ *b*). The tetramer has a square-planar shape with a disordered region of 114 residues located at the corners of the tetramer (Fig. 1 ▶ *b*). A comparison with the crystal structure of *S. pyogenes* IMPDH (PDB entry 1zfj; Zhang *et al.*, 1999 ▶) confirms this missing region as corresponding to the disordered CBS domain (Fig. 2 ▶ *a*). The structure of *Pa*IMPDH that we report is therefore consistent with other IMPDH structures (Hedstrom, 2009 ▶).

### 3.3. The active site

The active site of *Pa*IMPDH is located at the C-terminal end of the β-barrel, as is common for this (β/α)_8_-barrel fold (Hall *et al.*, 2002 ▶), and at the edge there is a flexible loop (residues 297–315; Fig. 2 ▶ *b*). This loop adopts different conformations at different stages of the catalytic cycle and is often disordered in crystal structures (Hedstrom, 2009 ▶). Recent studies have shown that the active-site loop controls the transition between the dehydrogenase and hydrolase activities (Josephine *et al.*, 2010 ▶). This loop is fully ordered in *Pa*IMPDH, with an average *B* factor of 33.6 Å^2^, which is comparable to the average for the whole protein (33.7 Å^2^), and adopts an ‘open’ conformation in contrast to the ‘closed’ conformation observed in other IMPDH structures (Fig. 2 ▶ *b*).

### 3.4. Potential for selective inhibition of *Pa*IMPDH

**Figure 3:**
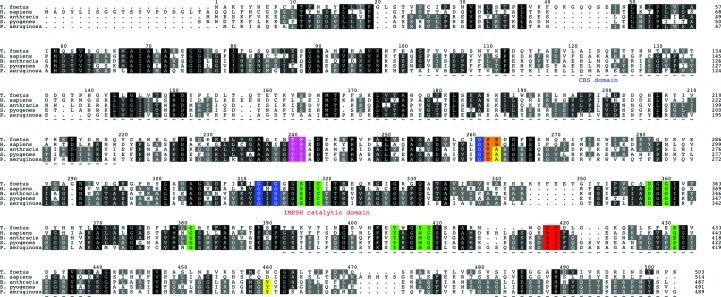
Sequence alignment of *Pa*IMPDH with homologues from *T. foetus* (GenBank ABI11203.1), *H. sapiens* (NCBI Reference Sequence NP_000875.2), *B. anthracis* (UniProt Q81W29) and *S. pyogenes* (UniProt P0C0H6). Residues involved in interactions with IMP, including the catalytic cysteine, are coloured green. Conserved residues interacting with NAD^+^ are coloured blue and nonconserved residues interacting with the adenosine and pyrophosphate portions are coloured magenta and orange, respectively. Two conserved residues on the mobile flap are coloured red. Residues involved in selectively binding the inhibitor C64 are highlighted in yellow. Residues missing in the *Pa*IMPDH structure have a dashed line beneath them. The sequence alignment was generated and annotated using *ClustalW* (Larkin *et al.*, 2007 ▶) and *ALINE* (Bond & Schüttelkopf, 2009 ▶), respectively.

**Figure 4:**
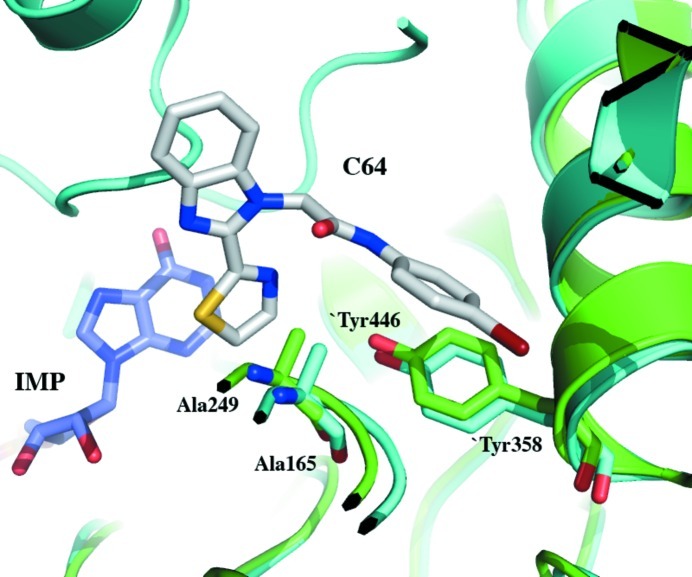
*Pa*IMPDH (green) superimposed onto the structure of *Cp*IMPDH (PDB entry 3khj; cyan) with the substrate IMP and the inhibitor C64 bound. Two residues involved in the selectivity of C64 towards the parasite enzyme IMPDH are shown and are also present in *Pa*IMPDH. Residues marked with a prime are from the adjacent subunit that forms the catalytic tetramer.

A sequence alignment of *Pa*IMPDH with selected homologues shows that the key residues involved in catalysis are conserved across species (Fig. 3 ▶). However, variation in the residues interacting with the adenosine and pyrophosphate portions of the NAD^+^ cofactor have been observed (Fig. 3 ▶) and are believed to be good targets for achieving selective IMPDH inhibition (Hedstrom *et al.*, 2011 ▶). Furthermore, *Pa*IMPDH possesses a structural motif that indicates that this enzyme belongs to a group of IMPDHs that are susceptible to inhibitors developed to target *Cp*IMPDH (MacPherson *et al.*, 2010 ▶; Hedstrom *et al.*, 2011 ▶). The compound *N*-(4-bromophenyl)-2-[2-(1,3-thiazol-2-yl)-1*H*-benzimidazol-1-yl]acetamide, labelled C64, is particularly relevant. This compound is a potent inhibitor of *Cp*IMPDH, with an IC_50_ of about 30 n*M*, but not of the human type II enzyme. Several residues have been identified that contribute to this observation (MacPherson *et al.*, 2010 ▶). Of particular importance are two residues that interact with the bromoaniline moiety of C64: Ala165 and Tyr358 (Gollapalli *et al.*, 2010 ▶; Fig. 4 ▶). The alanine and tyrosine residues create a hydrophobic surface that forms van der Waals interactions with the bromoaniline group. These residues are conserved in bacterial homologues, including *Pa*IMPDH (Ala249 and Tyr446; Figs. 3 ▶ and 4 ▶), but differ in the human enzyme (Ser276 and Asp470; Fig. 3 ▶). A further seven residues in *Cp*IMPDH are implicated in assisting C64 binding and these are Ser22, Pro26, Thr221, Ser354, Gly357, Met308 and Glu329 (data not shown). In *Pa*IMPDH these residues correspond to Gly19, Pro25, Thr306, Ala442, Gly445, Met393 and Glu417 (Fig. 3 ▶). This high degree of conservation extends to other bacterial homologues but not to the human enzyme (Fig. 3 ▶). This reinforces the idea that selectivity for the bacterial enzymes over human IMPDH is possible. With an efficient recombinant expression system for *Pa*IMPDH and established crystallization conditions, the scene is set for a structure-based approach, noting that the reagents and data generated in the study of protozoan homologues might now be exploited to fast-track the discovery of new IMPDH inhibitors relevant to antibacterial drug discovery.

## Supplementary Material

PDB reference: inosine 5′-monophosphate dehydrogenase, 3zfh

## Figures and Tables

**Table 1:** Crystallographic statistics for *Pa*IMPDH Values in parentheses are for the highest resolution shell.

Space group	*I*4
Unit-cell parameters (Å)	*a* = *b* = 115.5, *c* = 56.4
Resolution (Å)	19.1–2.25 (2.37–2.25)
No. of reflections recorded	86989 (12454)
Unique reflections	16612 (2476)
Completeness (%)	94.6 (97.0)
Multiplicity	5.2 (5.0)
〈*I*/σ(*I*)〉	16.3 (4.3)
Wilson *B* (Å^2^)	29.3
No. of residues	293
No. of waters	117
*R*_merge_ † (%)	9.1 (48.9)
*R*_work_ ‡ (%)	14.8
*R*_free_ § (%)	19.0
Average *B* factor (Å^2^)	
Protein	33.7
Waters	38.5
Chloride	72.1
Cruickshank DPI¶ (Å)	0.2
Ramachandran plot	
Most favoured (%)	97.3
Additional allowed (%)	2.0
Outliers (%)	0.7
R.m.s.d. from ideal values††	
Bond lengths (Å)	0.01
Bond angles (°)	1.5

† *R*_merge_ = 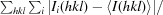 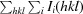, where *I_i_*(*hkl*) is the intensity of the *i*th measurement of reflection *hkl* and 〈*I*(*hkl*)〉 is the mean value of *I_i_*(*hkl*) for all *i* measurements.

‡ *R*_work_ = 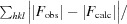 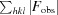, where *F*_obs_ is the observed structure factor and *F*_calc_ is the calculated structure factor.

§ *R*_free_ is the same as *R*_cryst_ except that it was calculated with a subset (5%) of data that were excluded from the refinement calculations.

¶ Diffraction precision index (Cruickshank, 1999 ▶).

†† Engh & Huber (1991 ▶).
